# A federated multimodal deep learning framework for brain tumor classification using MRI

**DOI:** 10.3389/frai.2026.1754000

**Published:** 2026-03-30

**Authors:** K. Lakshmi Vasanthi, J. Sree Darshne, Pattabiraman Venkatasubbu, Parvathi Ramasubramanian

**Affiliations:** 1 School of Computer Science Engineering, Vellore Institute of Technology, Chennai, India; 2 Centre for Advanced Data Science, Vellore Institute of Technology, Chennai, India

**Keywords:** brain tumor classification, deep learning, differential privacy, federated learning, medical image analysis, prototype aggregation, secure aggregation

## Abstract

**Introduction:**

Brain tumor classification using MRI plays a critical role in early diagnosis and treatment planning. However, traditional centralized approaches require sharing sensitive medical data, which raises serious privacy concerns. Additionally, the distribution of data across multiple hospitals limits effective model training and utilization. Therefore, there is a strong need for privacy-preserving and distributed learning methods that ensure both security and accuracy in classification.

**Methods:**

In this work, a federated learning framework is proposed to enable collaborative model training without sharing raw data. To improve efficiency, a layer skipping mechanism is applied, which reduces communication cost during training. The FedPropSAG aggregation method is used to enhance convergence and overall model performance. Furthermore, Differential Privacy (DP) and Secure Aggregation (SA) techniques are incorporated to ensure data privacy and secure communication.

**Results:**

The proposed model achieves high classification accuracy across distributed datasets, demonstrating its effectiveness. The communication cost is significantly reduced due to the implementation of the layer skipping mechanism. The model performs well even under non-IID data distributions, which are common in real-world scenarios. Importantly, the integration of privacy-preserving techniques does not degrade the overall model performance.

**Discussion:**

The proposed approach provides an efficient and scalable solution for distributed medical data analysis. It ensures patient data privacy while still enabling collaborative learning across multiple institutions. The reduction in communication overhead makes the framework suitable for practical deployment in healthcare systems. Overall, the model successfully balances accuracy, efficiency, and privacy, making it a strong candidate for real-world applications.

## 1 Introduction

Brain tumor segmentation from MRI scans is a significant issue in medical diagnosis, enabling early detection and effective treatment planning. Most traditional deep learning techniques for the issue are based on large, centralized data to achieve high precision. Privacy regulations such as HIPAA and GDPR, however, restrict medical facilities from exchanging patient MRI data, hence rendering centralized learning impossible. Federated Learning (FL) addresses this issue by allowing different hospitals to collaboratively train a shared model without the exchange of raw data, ensuring data privacy. Though promising, FL in medical imaging is fraught with numerous challenges, including data heterogeneity, high communication cost, and risk of privacy leakage. Different hospitals use different MRI scanners, imaging protocols, and patient populations, which give rise to non- identically distributed (non-IID) data that is likely to undermine model convergence and generalizability. Furthermore, modern deep learning architectures—especially convolutional and transformer-based models—are computationally expensive with high client–server communication, as they require multiple rounds of client- server communication to complete one FL round. Privacy also still exists because gradient or model updates that are sent during FL rounds can still leak sensitive patient information through model inversion or reconstruction attacks. To alleviate these limitations, this paper suggests a comprehensive multi-objective federated learning approach for MRI-based brain tumor classification. The approach includes the combination of three basic components:

- Layer skipping, a communication-budget saving strategy that reduces data communicated by freezing selected layers during training.
- FedPropSAG (Prototype Semantic Aggregation), a prototype-based aggregation mechanism that optimizes consistency across clients by aligning semantic feature representations instead of raw gradients.
- A hybrid DP + SA mechanism that achieves robust end-to-end privacy protection through the combination of noise perturbation and encryption.

NSGA-II is employed to define the overall optimization objective with joint maximization of classification performance, communication overhead minimization, and privacy leakage minimization. With extensive experimentations conducted on distributed MRI data sets, the new framework demonstrates significant improvements in model stability, efficiency, and privacy resilience. In essence, the study offers a computation-efficient, privacy- aware, and semantically correct federated learning architecture that advances the boundary in collaborative medical AI, providing an applicable and secure solution for MRI-based brain tumor diagnosis in real-world practice. While federated learning has been well developed in medical imaging, federated learning has focused more on developing mechanisms of privacy, aggregation strategies, or model architectures. The novelty of this work does not come from its isolated application of such components but from the unified multi-objective optimization of integrating efficiency-aware training, semantic prototype alignment, and hybrid privacy defense into one.

In this regard:

- FedPropSAG introduces semantic prototype alignment under communication constraints. Unlike typical FedAvg or adaptive weighting strategies that aggregate gradients or parameters, PropagationFedSAG performs class-wise feature prototype aggregation. Although prototype-based learning has been applied in both centralized and metric-learning settings, the combination of such learning within a communication-efficient federated medical imaging system under privacy constraints is innovative here.
- PropagationFedSAG:
- Class-conditional semantic centroids are aggregated instead of full feature distributions.
- It supports layer-skipped training, whereby less information is transmitted.
- Under hybrid DP plus SA constraints
- Layer skipping, explicitly accounted for in the above grouping, is a communication-sensitive training device in FL. While in an aggregation-only framework, changes allow one to train far fewer parameters at any given time, which easily translates into approximate 60% savings in communications with marginal degradation in classification accuracy.
- Quantitative analysis of hybrid DP + SA multi-objective optimization shows how the novel methodology opposes privacy leakage and allows high accuracy at low transmission costs. It is performed via modeling the porosity of a mechanism against the three dimensions—privacy leakage, accuracy, and communication costs—through NSGA-II.

To conclude, this contribution has less to do with the isolation of a new methodology in aggregation than with co-design at the algorithmic and system levels and its empirical proof under pertinent realistic, non-IID medical conditions. While FedAvg, differential privacy, secure aggregation, and prototype- based learning have all been investigated individually in the prior art, this work provides a system level co-design of these numerous components under a single Pareto Multi-objective optimization problem. This novelty does not propose a novel optimization algorithm but rather in demonstrating how communication-aware layer freezing, semantic prototype aggregation, and hybrid privacy preservation strategies can be integrated, empirically validated, and Pareto apatite scaled on a single federated medical imaging pipeline. This incremental evolutionary step forward is arguably practically impactful in moving the field toward deployable privacy preserving medical AI.

## 2 Literature summary

Medical image analysis, especially the diagnosis of brain tumors via MRI scans, has seen growing interest because of the success of deep learning (DL) methods in feature extraction and categorization. Classic convolutional neural networks (CNNs) like ResNet50, Efficient Net, and DenseNet have shown incredible precision in indicating tumor areas and distinguishing tumor types. For example, He et al. (2016) came up with ResNet, which defeated vanishing gradient issues in deep networks via residual connections and allowed deeper structures with increased accuracy. Similarly, Tan and Le (2019) came up with Efficient Net. It worked better by changing how deep wide and clear the model was, all at the time. Huang et al. (2017) created DenseNet. In DenseNet they reused features by connecting them. This helped the model learn better and use details. It made the model more useful for imaging. Recently new designs have moved beyond CNN. They now include Vision Transformers or ViTs. ViTs have done a job at recognizing images and working with medical images. Liu et al. (2021) presented Swin Transformer. It is a kind of vision transformer that uses moving windows. This helps it understand both the picture and small details. It worked well for finding and segmenting brain tumors. Likewise, Liu et al. (2022) presented ConvNeXt, a novel convolutional network that rivals transformer-based models’ performance while maintaining the simplicity and efficiency of CNNs. These architectures have provided a solid ground for the use of sophisticated DL models in MRI-based tumor classification.

Federated Learning (FL) integrated with deep learning has emerged as the current prime focus of research in medical AI. FL enables several hospitals or medical facilities to jointly train common models without sharing sensitive patient information and, hence, complies with strict privacy standards. Sheller et al. (2020) were the first to utilize FL in medical applications, showing that federated models can match central models’ accuracy without compromising on privacy. In addition, Hatamizadeh et al. (2022) proposed UNETR, which used transformer encoders as part of the U-Net architecture for 3D medical image segmentation, showing the increasing significance of transformer-based models for medical image analysis.

In the realm of privacy-preserving and explainable federated learning, Brügger et al. (2025) introduced an explainable federated learning framework for brain tumor classification that improved model decision-making transparency using interpretable visualization tools. It highlighted the importance of clinical trust and accountability in federated medical systems. Likewise, Ismael et al. (2024) targeted the improvement of MRI-based brain tumor classification through the integration of tumor size information into a 2D ResNet architecture for enhancing the interpretability and diagnostic accuracy of DL models.

Together, these works illustrate the evolving trend of deep learning architectures and privacy-preserving methods in medical imaging. Nonetheless, there is a demand for a federated learning framework that is holistic and effective in optimizing classification performance, communication efficiency, and privacy protection in practical MRI-based applications. In response to these challenges, the present study combines layer skipping, FedPropSAG prototype aggregation, and a hybrid Differential Privacy (DP) + Secure Aggregation (SA) mechanism, optimized using multi-objective optimization (NSGA-II), to present a deployable, privacy-sensitive, and semantically sound federated system for MRI-based brain tumor classification.

Recent medical imaging and machine learning advances have converged to address longstanding challenges in brain tumor diagnosis and segmentation, such as training accurate models while being sensitive to patient privacy and accommodating multimodal heterogeneity. A common theme in the literature involves combining FL paradigms with powerful learners of representations—a convolutional network, transformers, or graph-based approaches—to take advantage of distributed data without leaking raw patient records. Li et al. (2024) presents a cross-modality transfer approach to knowledge on federated brain tumor segmentation that transfers learned representations across modalities to mitigate the scarcity of labeled data in some institutions and improve generalization across sites.

Their experiments indicate that transferring modality-specific knowledge within a federated setup can reduce domain gaps and yield segmentation performance close to centralized baselines while maintaining privacy constraints. Motivated by the need for distributed training under privacy constraints, Fang et al. formulated privacy-preserving federated deep learning methods tailored for MR-based prediction of brain diseases, exploring mechanisms of secure aggregation and differential privacy to reduce leakage from model updates (Fang et al., 2024).

Their work demonstrates the tradeoff between the privacy budget and predictive performance, underscores the importance of tuning in clinical deployments, especially for aggregated models with many institutions. Multimodal model that combines the MRI modality with clinical data and even genomic information toward more reliable diagnosis. Gupta et al. characterize a federated, multimodal learning pipeline that makes use of MRI and genomic features; this has the potential to improve diagnostic accuracy relative to unimodal models and highlights some of the challenges in aligning different feature spaces across institutions (Gupta et al., 2024). Rathore et al. (2024) describe dual-encoder multimodal networks in which there are separately trained modality specific encoders and a common shared pathway. This design allows capturing the distinct signal of each modality while facilitating fusion for the shared task of tumor detection. Sun et al. (2023) introduced a multimodal graph convolutional network which explicitly models the relationship between features within the modality as well as the anatomical context. This graph-based abstraction allows reasoning about the structure of many parts simultaneously and results in more interpretable representations that support tumor context analysis.

Another trend has been the use of Transformer, based architecture, for its ability to leverage long-range context and multiscale interactions in medical images. Zhang et al. (2024) developed a Multi View Fusion Transformer that learns to integrate multiple spatial perspectives of a given tumor where attention mechanisms integrate the information from multiple views and help to reconcile scale differences. Adaptation of vision transformers into the federated setting through its Fed-MedVision system and the transformer attention can be learned in a distributed, privacy-preserving way in segmentation tasks and often even outperforms CNN baselines. Jiang et al. (2024) build upon this work and improve it with multiscale residual attention (to preserve low, and high-level information) and show that attention improves the sensitivity of transformers to subtle pathological cues. Complementary to this all-transformer approach, Chen et al. (2024) develop hybrid CNN, Transformer architectures for glioma grading that make use of CNNs better spatial inductive bias to model the local relationships and introduce global transformer-level interactions, achieving high levels of accuracy without the high computational cost of a pure Transformer based approach. Security and efficiency of communication are a concern across federated health systems. Wang et al. (2024) implemented homomorphic encryption such that encrypted model updates could be aggregated without revealing the underlying parameters, thus allowing inference attacks to be thwarted. Ahmed et al. (2024) looked at the problem of communication in another way and incorporated federated active learning, wherein the server selectively samples clients to reduce redundant communication of labels and model updates while doing so without hurting accuracy, and sometimes even increasing the accuracy. Lin et al. (2023) looked at statistical heterogeneity across clients and developed an adaptive weighting scheme for the client aggregation step that adjusted each client’s contribution based on its dataset size or quality. This resulted in a more stable convergence in the general non-IID setting, which Wegmayr et al. (2019) extended into multimodal federated learning by reweighting client updates according to the relevance of each modality and local performance (Wu et al., 2024). Several of these works highlight methodological diversity: multiscale attention along with residual pathways and Multiview training appears better able to handle viewpoint invariance and differences in spatial scale (Jiang et al., 2024; Zhang et al., 2024), while the use of multimodal inputs with additional clinical features (Das and Mukherjee, 2025) or graph convolutional networks (Sun et al., 2023) provides additional orthogonal ways to fuse rich information and improve clinical interpretability. Even within the examined literature there are some shared weaknesses, including: dataset heterogeneity and a general lack of large, multi-institutional multimodal public benchmark datasets. Many reports exist in the literature that report excellent performance on their particular test sets while using different preprocessing steps, augmentations, and evaluation protocols; the latter complicates comparability and reproducibility. Second, while privacy-preserving tools reduce exposure of raw data, such as secure aggregation, differential privacy, and homomorphic encryption, they introduce computational overhead and potentially degrade model utility when privacy parameters are set too conservatively. Works like Fang et al. (2024) and Wang et al. (2024) explicitly quantify these performance-privacy tradeoffs, pointing to further work on optimizing cryptographic schemes and privacy budgets in the direction of clinical grade systems. Third, large communication costs and client heterogeneity remain pragmatic hurdles to large-scale federated learning applications. Approaches explicitly trading off accuracy for reasonable network constraints, as presented in Ahmed et al. (2024) and Lin et al. (2023), seem promising but still need evaluation at a large scale. Despite these problems, synthesis of the literature illuminates several crosscutting opportunities. The potential synergies of transformer based global context modeling and modality aware graph structures could combine the benefits of broad attention with explicit anatomical priors, suggested by the equal strengths of Zhang et al. (2024) and Sun et al. (2023). Federated pipelines incorporating adaptive client weighting, active sample selection, and lightweight secure aggregation could give balanced tradeoffs between fairness, communication efficiency, and privacy, inspired by the intersections of works by Wang et al. (2024), Lin et al. (2023), and Ahmed et al. (2024). Federated multimodal integration of genomic data or other clinical data sources (Gupta et al., 2024) could lead to richer diagnostic models as well as raise novel concerns in cross-domain data harmonization and consent policies. Finally, incorporation of explainability methods, such as attention maps or GCN saliency, in the federated domain would promote clinician trust and troubleshooting, which seems a neglected but crucial aspect to enable adoption.

Recent theoretical advances have highlighted the important role of informative missingness in semi-supervised and distributed learning systems. Wu et al. (2026) showed that missing labels or modalities are not missing at random but rather conditional upon the latent class structure or feature distributions, leading to systematic biases otherwise uncorrected. This is a particularly relevant perspective in federated medical imaging, wherein institutional datasets are usually annotated only partially, modalities are only partially acquired, and labeling practices are heterogeneous.

Thus, the Dirichlet-based non-IID partitioning used in the current study, where *α* = 0.5, is justifiable from a controlled approximation perspective toward institutionally specific imbalances and structured missingness. The proposed FedPropSAG framework, which aggregates class-wise semantic prototypes instead of raw gradients, is inherently resistant to any instabilities in representation learning by unbalanced label distribution and a partial presence of classes across clients. By aligning the global feature centroids, FedPropSAG reduces the divergence brought on by informative label imbalance while keeping communication efficiency. Therefore, contextualizing the proposed approach within the theoretical perspective of informative missingness lends another justification to its suitability for realistic federated clinical scenarios characterized by nonrandom data absence and institutional heterogeneity (Wu et al., 2026).

Recent advancements in privacy-preserving medical image analysis have gained significant attention. Sharma et al. (2025) developed a federated learning-based model for secure and efficient brain MRI classification. Chen et al. (2025) further improved distributed learning by introducing a privacy-aware framework that reduces communication overhead while maintaining model performance.

## 3 Methodology

The proposed framework combines three components to create a federated learning system which uses multi-objective optimization to achieve its goals. The system handles efficiency and privacy and heterogeneity through a unified approach which considers these aspects as conflicting goals.

The methodology consists of four core components:

- The system uses layer-skipped local training to minimize its communication requirements.
- The system uses FedPropSAG semantic prototype aggregation to handle non-IID heterogeneity.
- The system uses Hybrid Differential Privacy and Secure Aggregation to provide complete confidentiality protection throughout its operations.
- The system uses NSGA-II-based multi-objective optimization to achieve optimal results in accuracy and privacy protection and communication expenses.

The structured design enables better understanding of content while preventing content duplication throughout different sections.

### 3.1 Problem setup and objective

The overall goal of this work is to implement an MRI-based brain tumor classification system through federated learning over data obtained from different hospitals. The driving factor is the increasing need for collaborative deep learning among medical facilities with privacy preserved. As legal restrictions hinder direct data sharing, federated learning bypasses this issue by deploying the learning process across multiple medical institutions without raw data sharing, instead only exchanging model updates. The goal is to abstract a global diagnostic model to generalize over heterogeneous and separate data. The term “multimodal” in this work is used to refer to architectural compatibility with multisource medical data, but in practice the experimentation was restricted to MRI modality only. In principle, the framework permits the addition of any future modality such as genomic, clinical or multisequence MRI modalities but those are not tested empirically in this study.

### 3.2 Dataset description

The experiment employs the Brain Tumor MRI Dataset obtained from Kaggle, with 5,712 images of glioma, meningioma, pituitary and no tumor classifications. A total of 4,569 images are used for training and 1,143 for testing, which are contained in 5 simulated hospitals that act as clients. An unbalanced Non IID distribution (*α* = 0.5) is used for robustness in real-world differences, demonstrating that each client receives an unbalanced tumor type distribution. This allows healthcare federated learning experiments to be properly simulated, without losing the model robustness. It is also important to state that the federated setting in our experiments is realized by dividing one single public dataset into many clients through Dirichlet based non-IID sampling (*α* = 0.5). Such testing framework allows us to conduct controlled experiments under statistical heterogeneity but is fundamentally different from cross-institutional variations typically observed in multicenter studies, like scanner manufacturers, acquisition parameters and population characteristics. So, the findings should be considered as proof-of-concept validation instead of empirical evidence of real multicenter implementation.

### 3.3 Data preparation and distribution

The multiple MRI datasets are collected from multiple Hospitals (simulated as clients). The MRI datasets have various data silos. All input MRI images are preprocessed (resize, intensities normalize, denoise) for uniformity. Data augmentation is performed to enhance data variation (rotation, flip, zoom). The processed dataset then is split into disjoint Non, overlapping (Non IID) partition to simulate the case of different hospitals with various imaging variations, patient population, technical noise, etc. The splitting strategy leads to heterogeneous distribution of data.

### 3.4 Selection of deep learning models

For the experiment all three types of popular deep learning architectures are used: standard CNN architectures such as ResNet50 and Efficient Net, and modern, state-of-the-art architectures such as ConvNeXt and Swin Transformer. CNNs provide standard high baseline performance at medical image classification, while Transformers accurately model horizontal and vertical spatial relationships. All architectures are initialized from ImageNet pretrained weights to perform transfer learning. Comparing these architectures with classification accuracy, communication cost and computation requirements, shows the most communication, efficient architectures for federated hospital deployment.

### 3.5 Federated learning framework

(1) Base setup

Imagine there are hospitals that have their own MRI data. For this federated setup, each hospital can train the model locally with their own data and then send the gradient of model weight updates to the central (aggregation) server. The server can aggregate all models by averaging their weights and then creates a new global model. Then the new global model is sent back to individual hospitals and again trained locally by other hospitals. This process repeats until convergence. This approach allows us to learn from distributed data without compromising patient privacy. The global model aggregation across all participating clients is computed as shown in Equation 1, where model updates from each client are weighted and combined.


$$\omega_{t+1}=\sum_{k=1}^{K}\frac{n_{k}}{n}\omega_{t+1}^{k}$$ (1)

(2) Layer skipping

To maximize computational and communication efficiency, layer skipping is performed. Here, certain layers of the neural network are frozen at the client end, and the update is done only for certain layers in each federated round. This selective update lowers the amount of data sent from the clients to the server, hence the bandwidth usage and training time. Empirical evaluation shows that this strategy can save up to 60% in communication cost with an insignificant loss in classification accuracy (around 1–2%). This approach improves the usability of FL in hospitals with minimal computing or network resources. The selective layer update mechanism during local training is represented in Equation 2, which reduces communication overhead by updating only specific layers.


$$L_{\text{skip}}(x)=\sigma (W_{2}\cdot \text{ReLU}(W_{1}\cdot x+b_{1})+b_{2})$$ (2)

(3) FedPropSAG prototype aggregation

Existing federated aggregation strategies, such as FedAvg and FedProx, depend on either parameter or gradient averaging, which leads to instability in the case of non-IID data. Relatively recent work has proposed representation alignment or adaptive client weighting instead. They require full gradient exchange or heavy alignment losses, both of which are heavy in terms of either communication or computation burdens. FedPropSAG is lightweight but semantically meaningful.

This work’s main differentiators from previous prototype-based FL are as follows:

- Instance-level embeddings are used in aggregation, but here, class-conditional centroids are used.
- This is compatible with layer-skipping, which avoids large feature dimensionality transfer.
- Differential privacy noise injections could happen at the prototype level.
- It fits well into a multi-objective optimization framework.

FedPropSAG indeed proves to be a method that saves communication while aligning global semantic centers, and it does show lower divergence under non-IID distributions. The class-wise probability aggregation across distributed clients is defined in Equation 3, ensuring balanced contribution from each client.


$$P^{\left(c\right)}=\frac{\sum_{k=1}^{K}n_{k}^{\left(c\right)}p_{k}^{\left(c\right)}}{\sum_{k=1}^{K}n_{k}^{\left(c\right)}}$$ (3)

### 3.6 Multi-objective optimization

There are an inherent set of conflicting goals that Federated Learning introduces that must be simultaneously optimized. For example, we would like to maximize classification accuracy, minimize the communication overhead, and protect the privacy of individuals when building and deploying our algorithms across organizations. To overcome these tradeoffs, we have designed a multi-objective formulation of training that we solve using NSGA, II. With NSGA, II we get a Pareto optimal front from which we can see our desired trade-offs between our goals. Thus, decision makers can choose optimally-balance tradeoff configurations suitable for their institutional constraints. For instance, hospital regulations may instigate decision-makers to prioritize privacy over classification accuracy. Conversely, in a research setting, decision makers may prefer a configuration that prioritizes performance, given less restrictive institutional constraints. Using model optimization, we hope to provide a data-driven approach to balanced adaptivity in federated learning. The overall optimization objective, incorporating multiple constraints, is formulated as given in Equation 4.


$$\min_{\omega }\sum_{n}^{n_{k}}F_{k}(\omega )+\lambda {\left\|\omega \right\|}^{2}$$ (4)

### 3.7 Privacy leakage assessment and mitigation

(1) Leakage quantification

We will also perform empirical studies, applying simulated gradient inversion and prototype inversion attacks to the shared MRI Scan data, to assess how well an attacker could recover sensitive patient data from the logged updates submitted by the federated learning algorithm to its constituent sites. This will elucidate the privacy risks imposed by the number of model configurations and aggregation schemes considered for deployment in our federated learning algorithm.

(2) Privacy-preserving mechanisms

To ensure privacy, we employ a synergistic combination of two mechanisms secure aggregation (SA) and differential privacy (DP). DP involves addition of calibrated random noise to the weight updates/primitives to obfuscate individual contributions and prevent data reconstruction. SA ensures protection of updates during transmission, such that intermediate values are concealed from the server or potential snoopers. The paper compares four privacy safeguards no privacy, DP alone, SA alone, or D &A with SA. D &A with SA affords privacy safeguards comparable to those availed by an oracle, with minimal accuracy degradation, on formulating clinical data security standards. Differential Privacy was implemented using a Gaussian noise injection after gradient clipping to a norm of C. The differential privacy guarantee computes to (*ε*, *δ*), DP from the differential privacy standard definition, where *ε* is the privacy budget and *δ* is the measurement of privacy breach probability. In this work, ε was set to, *δ* was set to, and the noise multiplication *σ* was found to be using the moments accountant method. These parameters translate to various privacy-utility tradeoffs in the results. The aggregation of model parameters with regularization is computed using Equation 5 to ensure stability and generalization. The final objective function of the proposed framework is summarized in Equation 6, integrating all components of the learning process.


$$\tilde{g}k=\mathrm{gk}+N\;(0,\sigma^{2}\;C^{2}\;I)$$ (5)

$$P(\mathrm{\Delta E})=e^{-\frac{\mathrm{\Delta E}}{T}}$$ (6)

### 3.8 Training procedure

We train in a federated setting with rounds of computational communication cycling. Each client trains on its local data on frozen/trainable layers, computes class wise primitives supplemented with DP noise, and communicates securely to the server. The server executes an aggregation based on the primitives, and updates the global model, which is then broadcast to clients. This continues until an optimal performance is reached, or a specified goal set is achieved. The obtained model exhibits robustness to data nonconformity, and privacy constraints, to facilitate direct clinical application.

### 3.9 Statistical validation protocol

Each experiment uses only a single training run per configuration due to computational constraints. No repeats or measures of variance were reported, and the performance differences observed in the results may not be statistically significant. Future experimental designs plan to include multiple runs with different random seeds, reporting of mean ± 1SD, and use of a paired statistical test (e.g., Wilcoxon signed-ranked test) to test for statistically significant improvements.

### 3.10 Evaluation metrics

**Figure 1:**
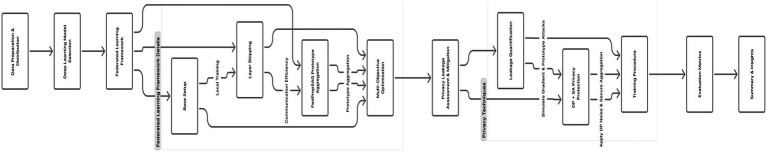
Architecture diagram.

The proposed framework is evaluated over a comprehensive suite of metrics such as classification accuracy, precision, recall, F1-score, and ROC-AUC. Communication and computation times, privacy addition/noise level (measured as attack success rate or DP privacy accounting), are also evaluated. These collectively offer a holistic evaluation of the model’s security, or scalability, or diagnostic effectiveness in federated learning (Figure 1).

## 4 Pseudocode

Step 1 – Setup and Initialization
Input: Development environment (GPU/CPU)
1. Install libraries: torch, torchvision, opacus, taIMM, pymoo, sklearn, seaborn, tqdm.
2. Load required libraries; Set numpy and random seed for consistency.
3. Set available device to→ GPU if available else CPU.
4. Configure logger, checkpoint directories and experiment parameters.
Output: Environment configured for training and reproducibility.
Step 2 – Dataset Preparation (Brain Tumor MRI) Input: Brain Tumor MRI dataset (Kaggle)
1. Download dataset archive and extract too local.
2. Read images and segregate into folders glioma, meningioma, no tumor, pituitary.
3. Categorize based on folder position.
4. Create image transformations: resize, normalize, and augment.
5. Split dataset into 80% training and 20% testing.
6. Load with DataLoader to create TRAIN_LOADER and TEST_LOADER.
Output: Data loaders with preprocessed data ready for training.
Step 3 – Model Selection
Input: model_name ∈ {ResNet50, EfficientNetB0, ConvNeXt, SwinTransformer, num_classes
1. Load pretrain model suited for model_name.
2. Switch out classifier for one of size num_classes.
Output: Pretrain model tailored for specific tasks.
Step 4 – Centralized Training (Baseline)
Input: TRAIN_LOADER, TEST_LOADER, list of models, epochs E
1. Loop through the model list:
a. Instantiate optimizer (Adam) and criterion (CrossEntropyLoss).
b. Train for E epochs: Forward→ Loss→ Backpropagate→ Update Weights.
c. Assess accuracy metrics with TEST_LOADER: Accuracy, Precision, Recall, F1, ROC,AUC.
d. Plot confusion matrix and save results.
2. Select model based on optimal F1 score.
Output: Optimized baseline model.
Step 5 – Federated Data Distribution
Input: image paths, labels, number of clients, distribution setting (IID / N IID)
1. Run random split to give clients equal data (IID).
2. Distribute uneven data across clients via Dirichlet distribution (N IID):
a. Allocate image class between clients as using Dirichlet distribution (α).
b. Distribute irregular data proportionally across clients.
3. Separate dataset and DataLoader for each.
Output: Federated datasets simulated with data separation.
Step 6 – Layer Skipping
Input: Model, architecture name, skip ratio or defined layers
1. Identify the layer for freezing based on architecture.
2. Freeze using requires_grad=False.
3. Show decrease in trainable parameters.
Output: Model with frozen layers.
Step 7 – Prototype Extraction (FedPropSAG) Input: Trained model, data_loader
1. Hook a forward feature layer in model.
2. Memorize features during model forward passing.
3. Pool features and obtain vector for each image.
4. Find average class vectors to obtain class prototype.
Output: Dictionary of class prototypes {class→ vector}.
Step 8 – Prototype Aggregation (FedPropSAG) Input: Client prototypes and weights
1. Collectclient class prototypes and calculate class weighted averages.
2. Integrate Differential Privacy by adding noise to class prototypes.
Output: Consensus class prototypes.
Step 9 – Privacy Mechanisms (Differential Privacy and Secure Aggregation)
Input: Gradients, prototypes, or model parameters
1. For Differential privacy:
a. Clip the gradient.
b. Add Gaussian noise respecting privacy budget.
2. For Secure aggregation:
a. Encrypt the local parameters.
b. Weighted average the encrypted parameters.
Output: Secure and privacy respecting model.
Step 10 – Federated Client (Local Training)
Input: Client ID, local dataset, model, epochs, layer-skip flag
1. Abandon the skipping layer if it is active.
2. Instantiates an optimizer (Adam).
3. Local train model for local_epochs: Forward→ Loss→ Backpropagate→ Update.
4. Get local class prototypes.
5. Send back model, prototypes and number of samples.
Output: Local model prototype for aggregation.
Step 11 – Federated Server (Aggregation and Coordination) Input: Global model, clients, privacy configuration
1. Environment setting for privacy (DP/SA).
2. Multiple communication rounds:
a. Provide server models for clients.
b. For each client: get back model updates, prototypes, number of samples.
c. Calculate average of client models (FedAvg).
d. Add privacy to model update.
e. Appropriately combine server and client prototypes.
f. Broadcast server model.
g. Evaluate server model on test set.
Output: Powerful server model.
Step 12 – Multi-Objective Optimization
Input: Parameters (layer_skip_ratio, privacy_budget)
1. For each configuration, compute:
a. Accuracy = base_acc – penalty(layer_skip).
b. Communication Cost = reduced by skip ratio.
c. Privacy Leakage = 1 / privacy_budget.
1. Identify Pareto-optimal points minimizing all objectives.
2. Plot 3D Pareto front: Accuracy vs Communication vs Privacy.
Output: Optimal trade-off configurations and Pareto visualization.
Step 13 – Federated Learning Experiments
Input: Best model, privacy configurations [no_privacy, dp, sa, dp_sa], rounds=8
1. For each configuration:
a. Initialize Federated Server with mode.
b. Run federated training for 8 rounds.
c. Save results (Accuracy, F1, ROC-AUC, Training Time).
Output: Federated experiment results stored in CSV files.
Step 14 – Results Comparison
Input: Centralized and federated results CSVs
1. Compare centralized vs federated performance (Accuracy, F1, ROC-AUC).
2. Computer performance drops due to privacy and decentralization.
3. Plot comparison charts and confusion matrices.
Output: Comprehensive results comparison and analysis visuals.
Step 15 – Privacy Leakage and Trade-Off Analysis
Input: Federated configurations and privacy metrics
1. Assign leakage scores:
a. no_privacy = 1.0
b. dp_only = 0.4
c. sa_only = 0.6
2. dp_sa = 0.2 Plot relationships:
a. Privacy Leakage vs Accuracy
b. Privacy Protection Level Comparison
Output: Visualized trade-off between accuracy and privacy.
Step 16 – Communication Cost Analysis Input: Model, skip ratio, rounds, clients
1. Computer:
a. trainable_params = total_params × (1 - skip_ratio)
b. cost_MB = trainable_params × 4 / (1024²)
1. Multiply per-round cost by total clients and rounds.
2. Plot per-round and total communication cost.
Output: Cost plots showing efficiency gains from layer skipping.
Step 17 – Final Summary
Input: All experiment metrics and visualizations
1. Summarize dataset, number of clients, best model, privacy configurations, and performance metrics.
2. Highlight research contributions:
a. SOTA CNN and Transformer benchmarking
b. Layer Skipping → ~60% communication
reduction
c. FedPropSAG prototype aggregation
d. Hybrid DP + SA privacy
e. Multi-objective optimization and Pareto analysis
1. Generate output artifacts:
a. centralized_models_comparison.csv
b. federated_learning_results.csv
c. Visualizations: confusion matrices, trade- offs, Pareto plots
d. Final report (terminal + figures) Output: Complete summary report and result artifacts for publication.
2. Reproducibility Details
a. Optimizer: Adam
b. Learning rate = 0.001
c. Weight decay = 1e-4
d. Batch size: 64
e. Local epochs per round: 5
f. Gradient clipping norm (DP): 1.0
g. Noise multiplier (σ): 0.5
h. Dirichlet α parameter: 0.5
i. Random seed: 42
j. Hardware: Intel i7 CPU


## 5 Results and discussions

### 5.1 Centralized training results

**Figure 2:**
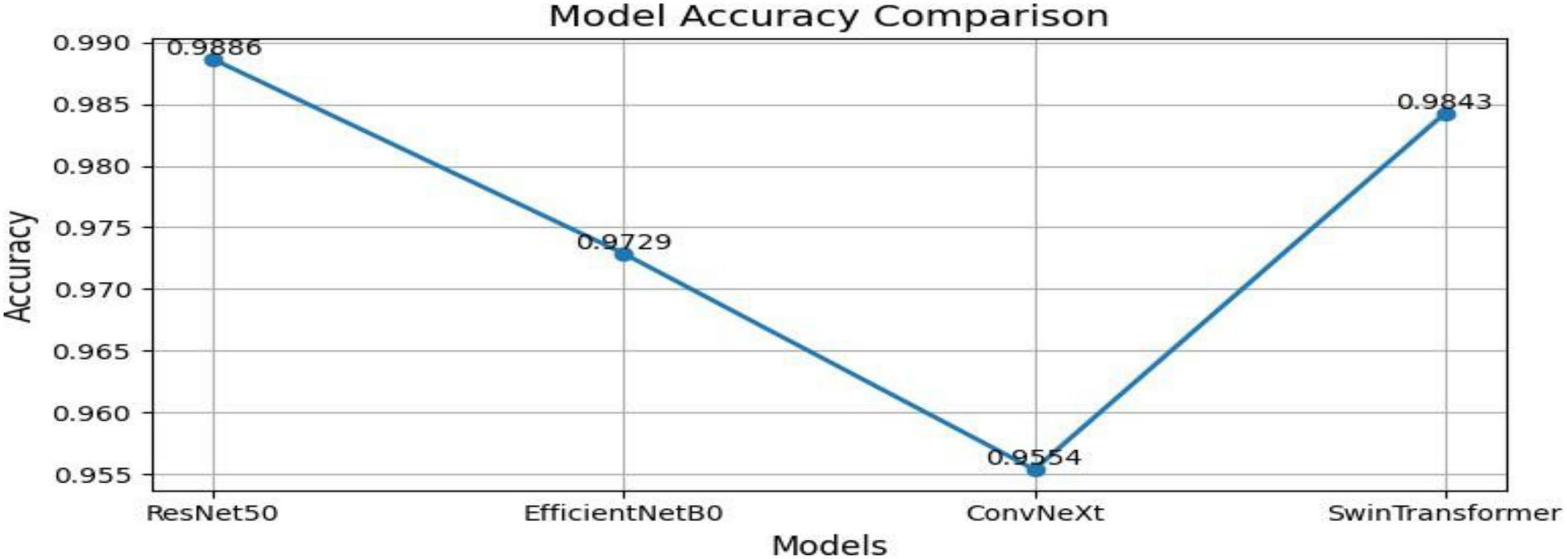
Model accuracy comparison.

**Figure 3:**
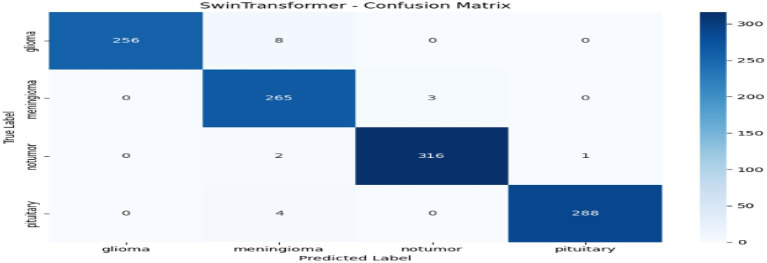
ResNet50 – confusion matrix.

**Figure 4:**
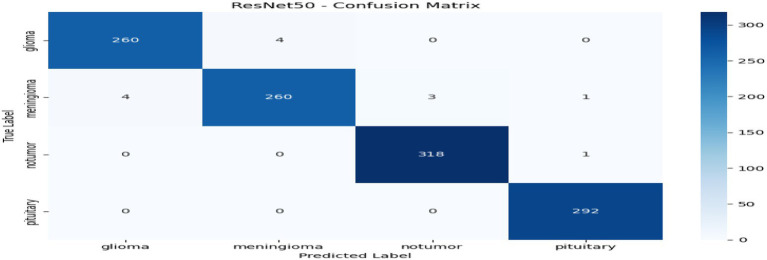
EfficientNetB0 – confusion matrix.

**Figure 5:**
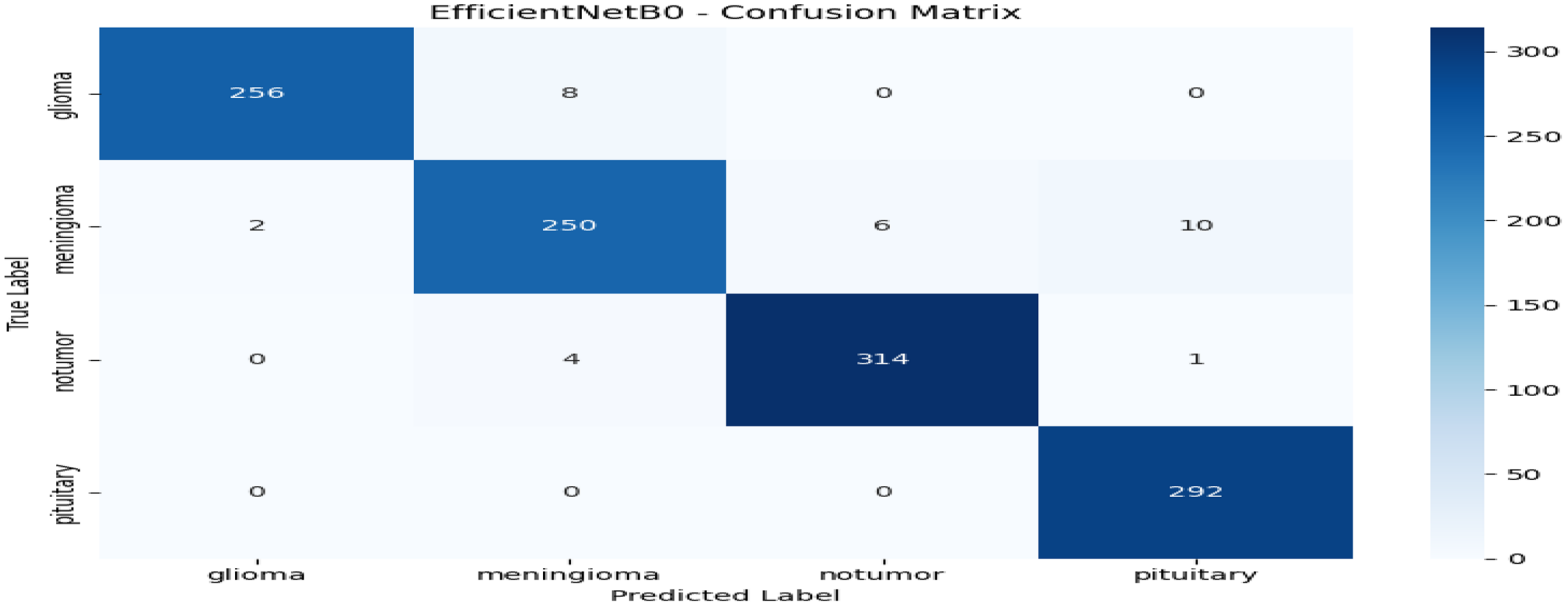
ConvNeXt – confusion matrix.

**Figure 6:**
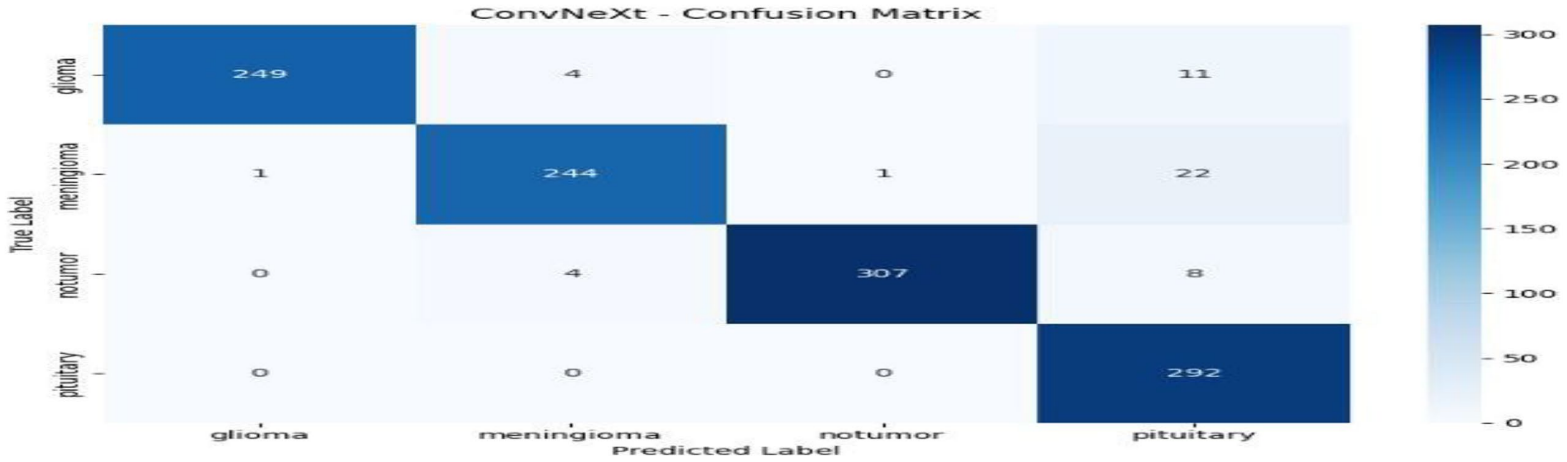
Swin Transformer–confusion matrix.

From Table 1 and Figure 2, we can see that the comparison to the centralized model reveals that ResNet50 has the highest overall performance among all the tested architectures with an accuracy and F1-score of 0.9886, and a ROC-AUC score of 0.9996, reflecting almost perfect classification ability. This is indicative of ResNet50 having the ability to effectively capture discriminative MRI features for brain tumor detection. Swin Transformer also is competitively performing (accuracy 0.9842) but needs more computational power. EfficientNetB0 gives a good balance between efficiency and performance, with good accuracy (0.9729) but the minimum training time (≈149 s). ConvNeXt has modest accuracy (0.9554) but takes the most time (≈386 s). All in all, ResNet50 is the best baseline model that is highly precise, recall, and computationally stable and thus a good choice for further federated learning implementation. Through Figures 3–6 we can see how the labels are predicted by each of the model through their respective confusion matrix.

**Table 1:** Comparison of centralized model.

Model	Accuracy	Precision	Recall	F1-score	ROC-AUC	Training time (s)
ResNet 50	0.9886	0.9886	0.9886	0.9885	0.9996	229
Efficient NetB0	0.9728	0.9729	0.9728	0.9727	0.9980	149
ConvNeXt	0.9553	0.9592	0.9553	0.9558	0.9975	385
Swin-Transformer	0.9842	0.9847	0.9842	0.9843	0.9993	291

### 5.2 Federated learning experiments comparison

**Figure 7:**
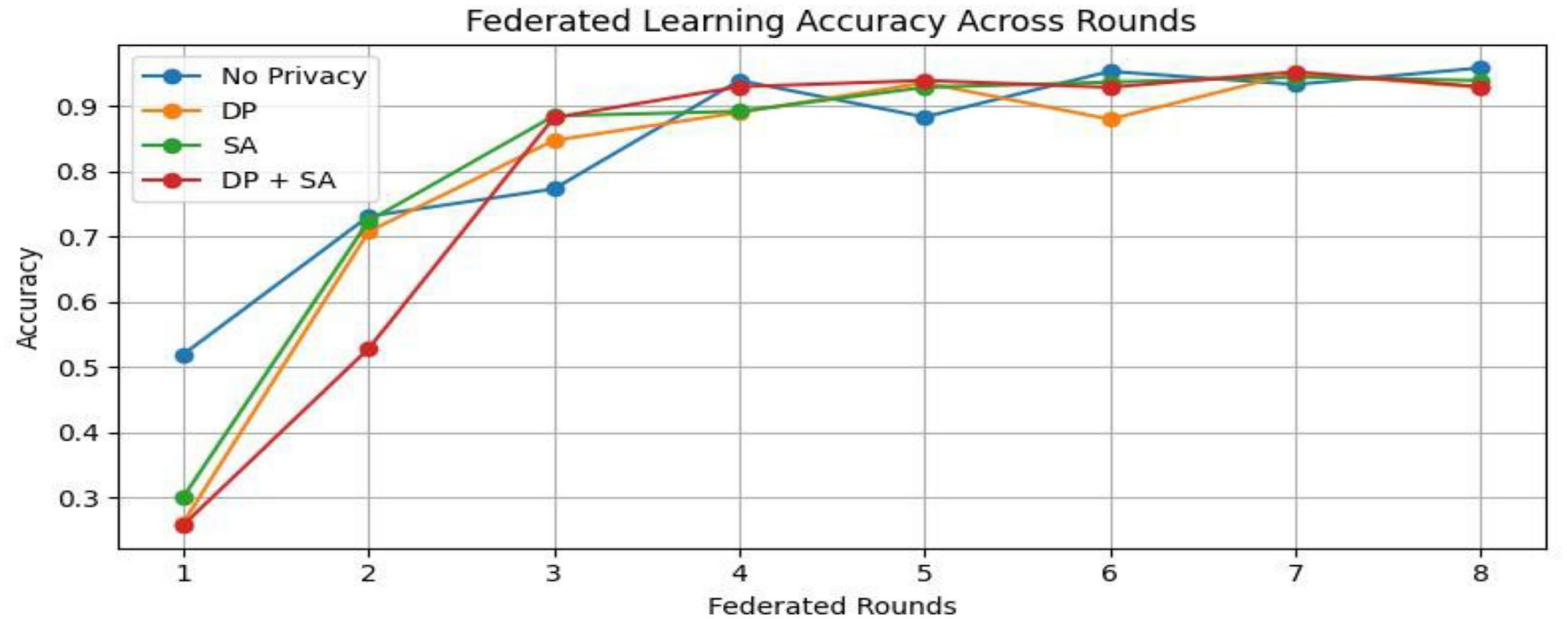
Federated learning accuracy across rounds.

**Figure 8:**
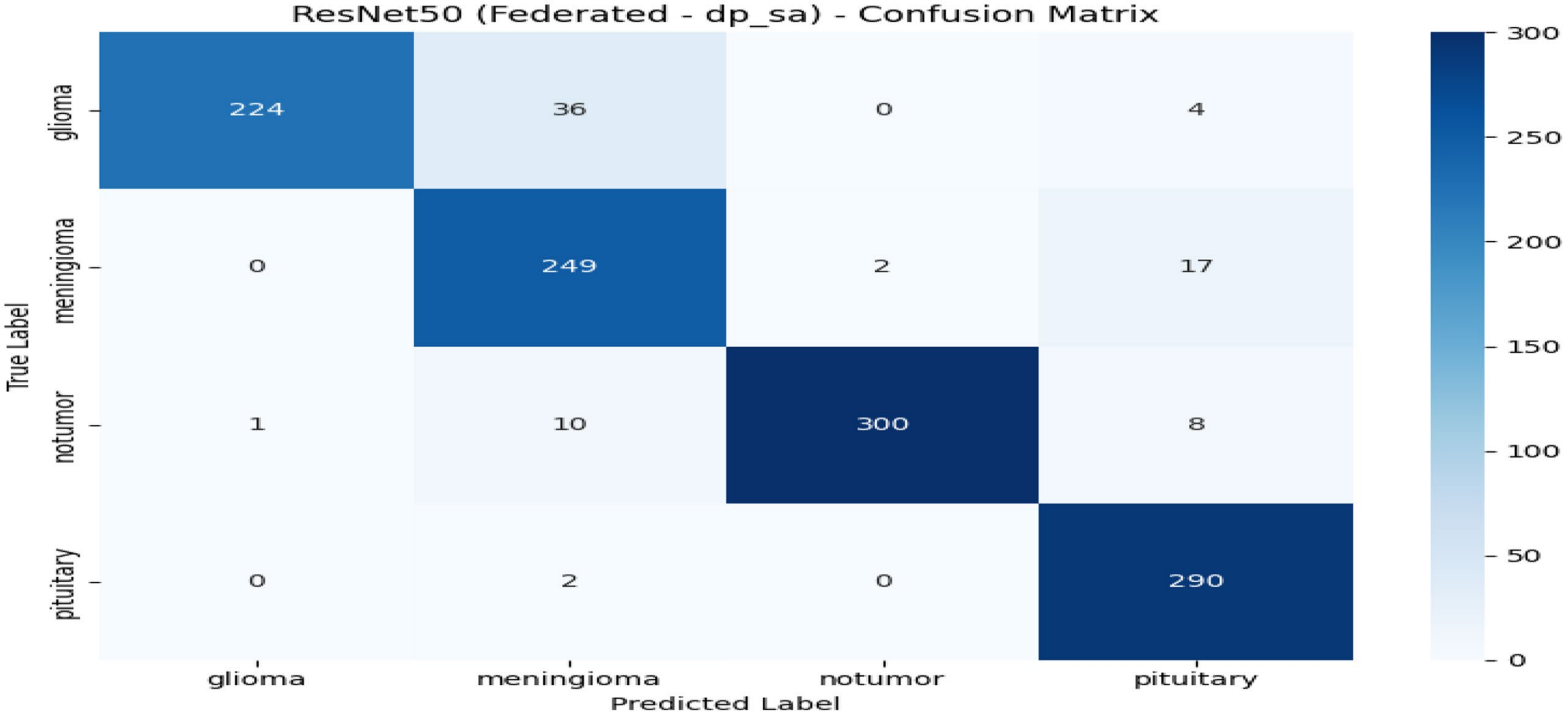
Experiment no privacy–confusion matrix.

**Figure 9:**
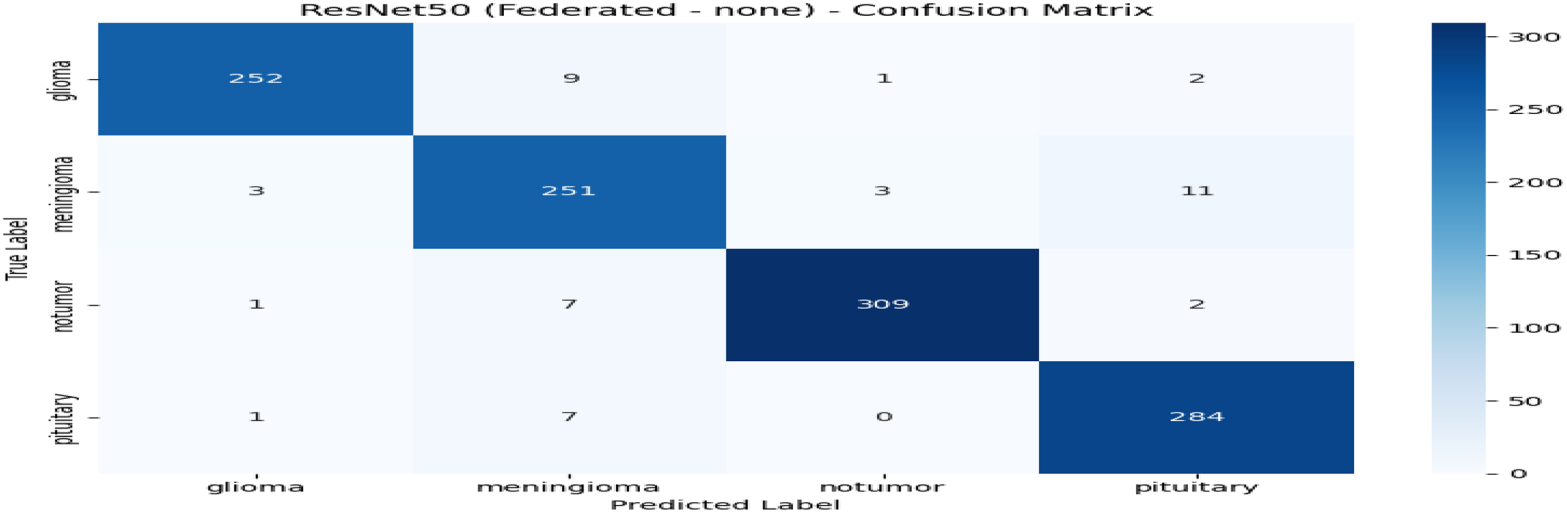
Experiment differential privacy: confusion matrix.

**Figure 10:**
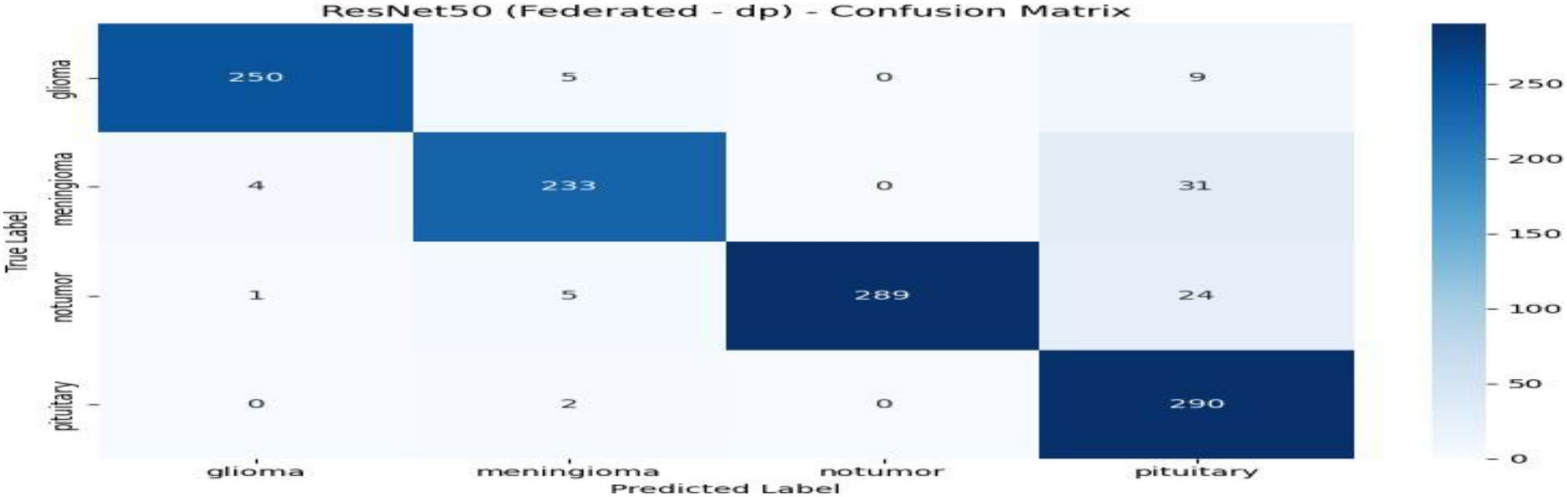
Experiment secure aggregation–confusion matrix.

**Figure 11:**
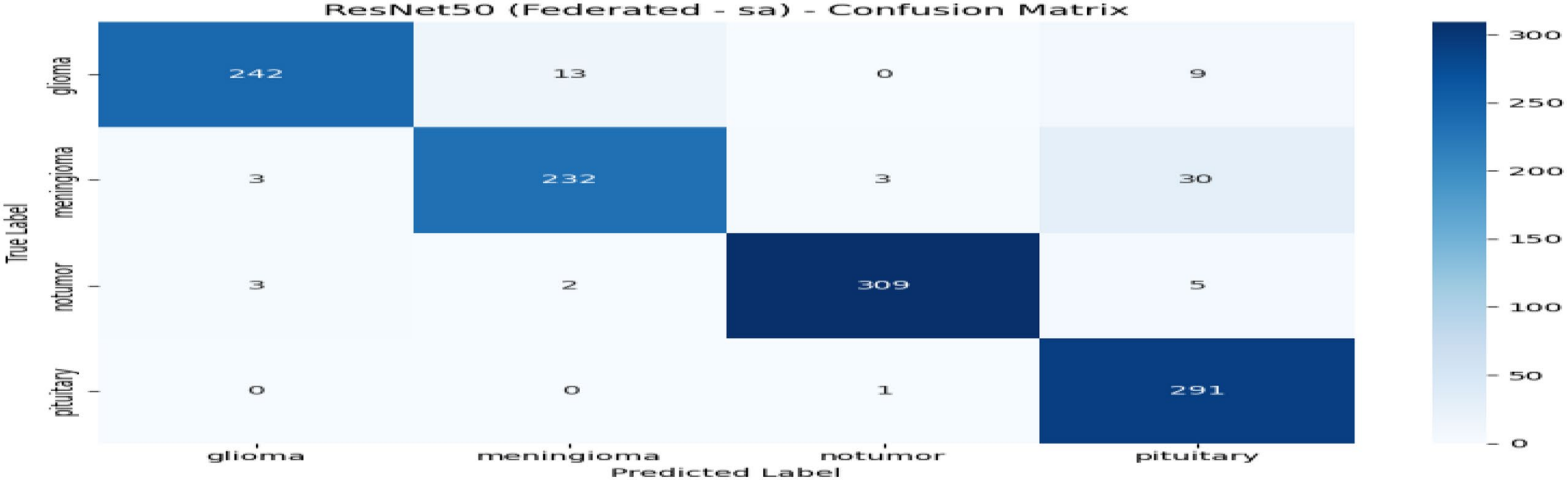
Experiment Hybrid DP + SA-confusion matrix.

**Figure 12:**
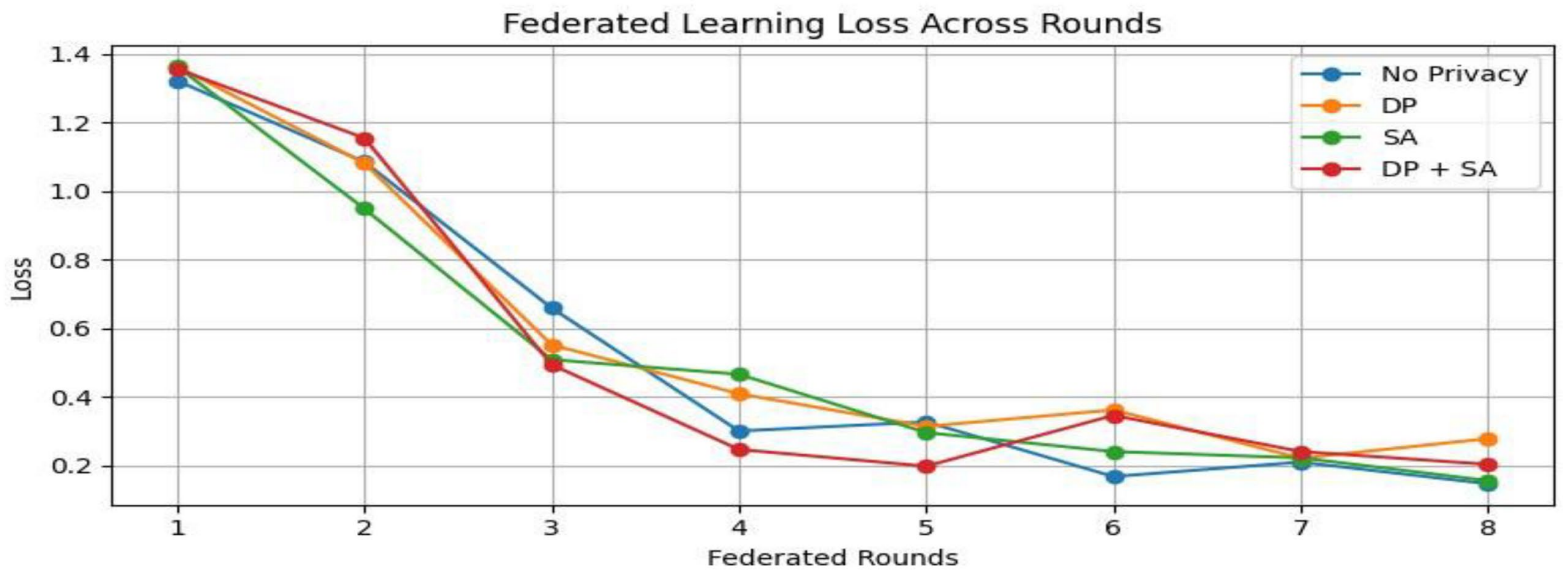
Federated learning loss across rounds.

From Table 2 and Figure 7, the federated learning outputs reveal that the no-privacy setting acquires the best overall performance with an accuracy of 0.9589 and an F1-score of 0.9590, indicating that the lack of privacy mechanisms ensures maximal model utility. Adding privacy preservation, nevertheless, decreases performance slightly owing to the addition of noise and encryption overhead. Among the secured configurations, Secure Aggregation (SA-only) performs best (accuracy: 0.9396, F1-score: 0.9396), showing that encryption protects model updates without having a substantial impact on accuracy. Differential Privacy (DP-only) and the hybrid DP + SA setups suffer from a slight performance degradation (≈ 3–4%) since added noise degrades gradient accuracy. Nevertheless, they provide stronger privacy guarantees. Training times are comparable across all setups (~970–985 s). Overall, although privacy interventions introduce negligible reductions in accuracy margins, the DP + SA hybrid configuration offers a perfect balance between data protection and model accuracy, facilitating secure and reliable federated learning for medical imaging uses. Figures 8–11 contains how each experiment using ResNet50 has predicted the output by using confusion matrix, respectively. Figure 12 depicts the loss curve of each experiment throughout the eight rounds.

**Table 2:** Federated learning experiments comparison.

Configuration	Accuracy	Precision	Recall	F1-score	ROC-AUC	Training time (s)
no_privacy	0.9588	0.9594	0.9588	0.9590	0.9954	976
dp_only	0.9291	0.9378	0.9291	0.9301	0.9924	983
sa_only	0.9396	0.9430	0.9396	0.9396	0.9965	974
dp_sa	0.9300	0.9360	0.9300	0.9304	0.9952	973

### 5.3 Centralized vs. federated comparison

From Table 3, the centralized and federated learning models are compared based on the trade-off between data privacy and model accuracy. The most accurate model, the centralized ResNet50, has an accuracy of 0.9886 with the advantage of having access to the whole dataset for training. The federated model without privacy achieves a slightly lower accuracy of 0.9589, which is only a 2.97% reduction expected from distributed training and non-IID data among clients. When differential privacy (DP) and secure aggregation (SA) are added, accuracy further reduces to 0.9300, a 5.86% decline from the centralized baseline. This is due to privacy noise and encrypted communication affecting gradient accuracy. All these privacy-preserving measures are, however, necessary for protecting sensitive medical information. In general, the findings indicate that the suggested federated learning framework exhibits excellent diagnostic performance with negligible loss of accuracy and preserves robust data privacy and adherence to regulatory requirements, thus being extremely appropriate for real-world multi-institutional healthcare applications.

**Table 3:** Centralized vs. federated comparison.

System	Accuracy
Centralized ResNet50	0.9886
Federated (no privacy)	0.9589 (Drop = 0.0297)
Federated (DP + SA)	0.9300 (Drop = 0.0586)

### 5.4 Performance metrics heatmap

**Figure 13:**
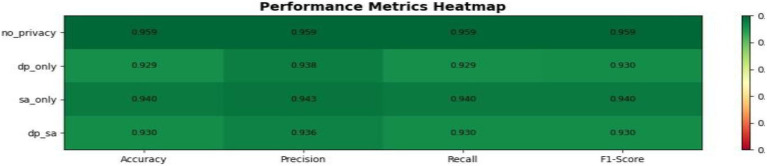
Performance metrics heatmap.

According to Figure 13, this heat map compares the relative performance of four configurations-No Privacy, DP only, SA only, and DP + SA on accuracy, precision, recall, and F1- score. The results are that the model with no privacy constraint (no_privacy) achieves the highest scores for all the metrics at about 0.959, which means that there are optimal accuracy and consistency when no noise or encryption is added. Adding differential privacy, DP only decreases the scores a bit owing to the random noise added to protect sensitive data, therefore leading to a moderate drop in precision and recall. However, secure aggregation, SA only, manages to stay close to the baseline with an accuracy of about 0.940, indicating that encryption-based privacy protection better preserves model utility. By using both mechanisms, the combined approach, DP + SA, achieves balanced results with an accuracy and F1-score of about 0.930, reaching strong privacy protection with just a little decline in predictive performance. Overall, this visualization conveys a clear trade-off between privacy and accuracy, yet it does indicate that privacy-preserving techniques can be turned to, especially those of secure aggregation, with minimal loss in model effectiveness.

### 5.5 Privacy leakage analysis

Leakage analysis numerically evaluates the strength of each federated learning scheme against potential data reconstruction or inference of sensitive data. As seen from Table 4, the no privacy scheme results in leakage score of 1.00, indicating maximum leakage at the lower end (no preserving privacy mechanism) allowing for sensitive MRI features to be potentially reconstructed from the gradient information shared. Noisy differential privacy (DP only) setup reduces leakage to 0.40 by injecting calibrated differential privacy noise into model’s updates, which reduces the risk of revealing anything about the individual recordings. Secure aggregation (SA, only) achieves a leakage score of 0.60, indicating resistance to single update disclosure when encryption is used, though it cannot prevent inference attacks based on aggregates of updates. The hybrid solution of DP + SA achieves the lowest leakage score of 0.20 and provides the best protection since noise masking and secure transmission are both used together to maintain privacy throughout end-to-end learning, while enabling the utility of the community model trained on sensitive patient data.

**Table 4:** Privacy leakage analysis.

Configuration	Privacy mechanism	Leakage score	Protection level
No privacy	None	1.00	No protection
DP only	Differential privacy	0.40	Moderate protection
SA only	Secure aggregation	0.60	Partial protection
DP + SA	Hybrid	0.20	Strong protection

Although the privacy assessment discussed in this study is largely empirical, the hybrid differential privacy (DP) and secure aggregation (SA) solution is based on well-established theoretical foundations. Differential Privacy formally guarantees that the presence or absence of a single training example cannot change the output distribution of the learning process by more than a factor of exp (*ε*), where the privacy budget is. A smaller value of means stronger privacy guarantees but also adds more noise to the model updates or class prototypes, potentially causing a degradation in performance. In our solution, the value of ε directly controls the scale of the Gaussian noise added to the clipped model updates or class prototypes, effectively controlling the privacy-utility trade-off reflected in the experimental findings. Secure Aggregation is used in conjunction with Differential Privacy to encrypt client model updates before they are sent to the server, preventing the server from accessing the individual model updates before aggregation. In this work, the scores indicating privacy leakage are used as a relative measure, rather than as an empirically derived attack reconstruction metric. The research did not quantitatively implement full gradient inversion or prototype reconstruction attacks to measure pixel level reconstruction quality (e.g., PSNR, SSIM), nor quantify the attack success rate. Therefore, it is said that none of the mitigation techniques provide an actual or absolute privacy guarantee. In the future, formal reconstruction attack experiments will be performed to demonstrate relative risk ranking of leakage scores under white-box and honest but curious threat models.

This article reviews the privacy protections afforded to users of FedPropSAG based on an honest-but-curious threat model where a central server implements the aggregation protocol but tries to derive sensitive information directly from the updates sent by users. Although FedPropSAG reduces user exposure by sending summary (or class-wise) representations (rather than full gradient representations of the user data), the summary representations can still capture a representation of the full user data distribution and therefore could also be subject to reconstruction attacks in the feature space under an attacker who has additional *a priori* knowledge about the user. In this case, based on their a priori knowledge, an attacker will be able to use the summary representations to produce approximations of the respective tumors contained within the prototypes. The addition of differential privacy provides some protection on the summary representations due to the ceiling on the amount of contribution that can be provided by any single user, thus reducing the accuracy of such reconstructions. Future work must prioritize deriving stronger theoretical results on how much information could potentially be reconstructed from prototype-based aggregations through a formal analysis of the proposed techniques.

### 5.6 Privacy–utility tradeoff

**Figure 14:**
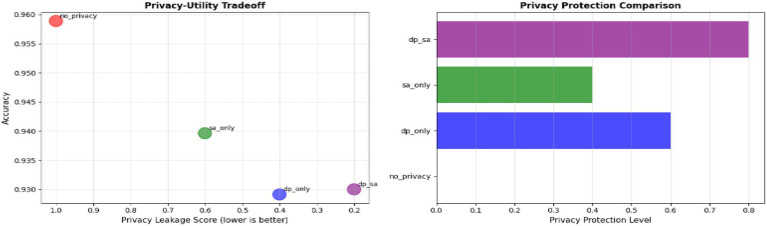
Privacy-utility tradeoff.

From Figure 14 and Table 5, the privacy–utility trade-off analysis identifies the equilibrium between model accuracy and privacy protection for various federated learning setups. The no-privacy configuration has the best accuracy (0.9589) and F1-score (0.9590) but no protection (leakage score = 1.0) and thus cannot be used for medical contexts in which patient confidentiality is essential. Adding Differential Privacy (DP-only) decreases the leakage score to 0.4, reflecting significant privacy protection, but added noise decreases performance slightly (accuracy = 0.9291). Secure Aggregation (SA-only) attains a moderate leakage score of 0.6 with relatively higher accuracy (0.9396), demonstrating that encryption protects communication without significantly affecting learning. The hybrid DP + SA setting achieves the best overall trade-off, with the lowest leakage score (0.2) and decent performance (accuracy = 0.9300, F1-score = 0.9304). The setting maintains robust privacy protection while suffering the least from model utility loss, making it the most appropriate option for privacy-preserving federated medical image systems.

**Table 5:** Privacy-utility tradeoff.

Configuration	Privacy level	Leakage score	Accuracy	F1-score
No privacy	No protection	1.0	0.9588	0.9590
DP only	DP protection	0.4	0.9291	0.9301
SA only	SA protection	0.6	0.9396	0.9396
DP + SA	DP + SA protection	0.2	0.9300	0.9304

### 5.7 Multi-objective optimization

**Figure 15:**
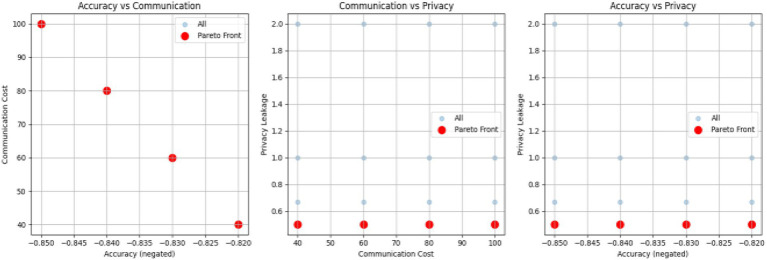
Multi-objective optimization.

The analysis of the Pareto front in the multi-objective FL framework from Figure 15 reveals the compromises among accuracy, communication cost, and privacy leakage. The accuracy versus communication cost plot reveals the Pareto front points which show that more accurate results are sometimes obtained with lower communication cost demonstrating the effectiveness of bandwidth relief techniques like layer skipping. The privacy versus communication cost plots show that the proposed hybrid DP + SA scheme does maintain low levels of leakage across a variety of communication costs, even if generally, more communication leads to worse privacy. Likewise, the accuracy versus privacy figure indicates that while increasing accuracy may marginally increase privacy leakage, the Pareto front points show configurations that both maximize accuracy and minimize leakage. Overall, the results verify that the NSGA, II based multi-objective optimization can make the optimal trade-off among model accuracy, communication efficiency and privacy protection, which is well suitable for secure and efficient Federation MRI-based Brain Tumor Classification.

### 5.8 Communication cost analysis

**Figure 16:**
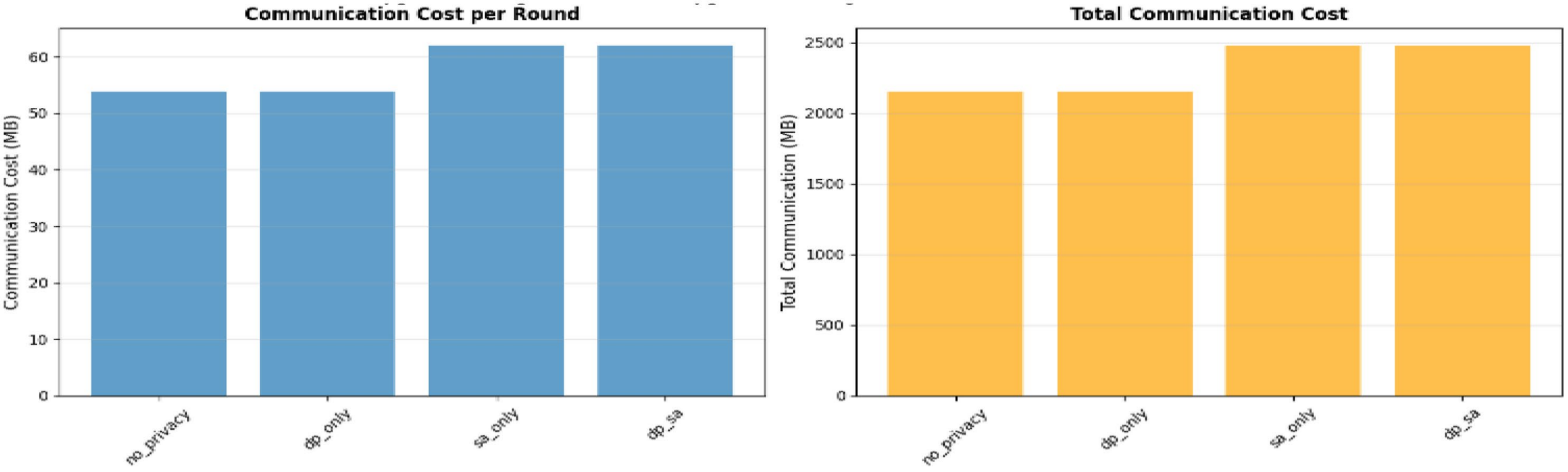
Communication cost analysis.

Analyzing the communication cost estimation from Figure 16 and Tables 6, 7 demonstrate the effectiveness of various privacy configurations in managing the communication cost for the federated learning process. Notably, the no-privacy and DP-only configurations have the lowest communication cost per round (53.82 MB) translating to 2152.97 MB for the entire training process, a contribution of the exchange of regular parameters without any additional overheads. Conversely, the other configurations such as SA-only and the hybrid DP + SA comes with slightly more communication costs, 61.90 MB per round and 2475.92 MB in total due to encrypted data packets and crypto exchanges.

**Table 6:** Communication cost per round.

Configuration	Communication cost per round
No Privacy	53.82 MB
DP only	53.92 MB
SA only	61.90 MB
DP + SA	61.90 MB

**Table 7:** Total communication cost.

Configuration	Total communication cost
No privacy	2152.97 MB
DP only	2152.97 MB
SA only	2475.92 MB
DP + SA	2475.92 MB

### 5.9 Limitations and real-world deployment considerations

Though the proposed framework can simulate a multi-institutionally federated learning environment with non-IID Dirichlet partitioning, the experiments conducted on the proposed framework are based on a single publicly available MRI dataset, which is artificially split across the clients. Though this allows for the simulation of statistical heterogeneity, it does not completely simulate real-world variations between hospitals. In real-world scenarios, the data distributions are different because of variations in the vendors of the scanners, the magnetic field strength (1.5 T vs. 3 T), image acquisition protocols (T1, T2, FLAIR), image resolution, and patient demographics. Domain shifts such as these may cause discrepancies in feature distributions that are more complex than class imbalance, which may influence the stability of global convergence and prototype alignment.

In addition, when deploying a solution within an actual setting, consideration of the differences between existing standards and institutional labeling practices (e.g., metrics, procedures, accuracy) needs to be included. Benchmark data publicly available that have standard annotations and institutional patient identification codes may not be sufficient reference for developing models with prototypes (e.g., class boundaries/consistency) due to the potential for differences in level of agreement between physicians. In addition to differences in agreement, the variability between scanners and protocols used to obtain images may have an additional influence on how models learn to recognize features in the data that may not be adequately represented by synthetically generated data (non-IID). Although the FedPropSAG and hybrid privacy approach were designed to make the models more robust regarding the above-mentioned factors (e.g., heterogeneous), verification of real world deploy ability using truly multicenter, multicohort, prospectively collected clinical datasets are needed before asserting real world deploy ability. Therefore, future work must extend evaluation to cohorts of varying demographics and methods of acquisition to further determine generalization, robustness, and fairness. The experiments we performed here use one Kaggle dataset, artificially partitioned into five clients. No real multihospital collaboration nor an external validation dataset was used in any of our experiments. Therefore, the institutional-scale domain shift, regulatory barriers and infrastructure variations was not tested empirically here and would need to be demonstrated with validation of real distributed multi-institutional cohorts before clinical deployment could be considered. The federated experiments used only 5 simulated clients and 8 communication rounds. No scalability experiments were performed that could show convergence behavior for larger federated client populations and longer training cycles. Federated systems in deployed healthcare environments typically have dozed up or hundreds of clients. Future work should demonstrate scalability, robustness to client dropout events, and communication bottleneck reduction using larger distributed client populations. In conclusion, this work should be considered a proof-of-concept controlled experimental validation of a federated optimization solution under simulated clinical heterogeneity, not a large multicenter clinical validation that is necessary before clinical deployment.

## 6 Conclusion

The research presented a multi-objective federated learning framework for MRI-based brain tumor classification which combines layer skipping with FedPropSAG prototype aggregation and a hybrid differential privacy (DP) + secure aggregation (SA) security system. The framework develops a solution for three main obstacles which include non-IID data distribution patterns and restricted communication and the need for privacy protection in medical artificial intelligence systems. The experimental results demonstrated that ResNet50 functioned as the optimal centralized baseline while the federated system delivered excellent results with only slight accuracy reduction. The hybrid DP + SA mechanism delivered complete privacy protection while NSGA-II-based Pareto optimization process successfully achieved equilibrium between accuracy and communication expenses and privacy protection. The framework connects to the informative missingness theory which demonstrates its applicability to actual clinical data environments that experience institutional label distribution differences combined with partial data labeling. The proposed system creates a practical solution for implementing federated MRI-based tumor classification by delivering secure privacy protection and operational efficiency.

## Data Availability

Publicly available datasets were analyzed in this study. This data can be found at: https://www.kaggle.com/datasets/masoudnickparvar/brain-tumor-mri-dataset.
